# Trends in Prevalence of Insulin Resistance Among Nondiabetic/Nonprediabetic Adolescents, 1999–2020

**DOI:** 10.1155/pedi/9982025

**Published:** 2025-05-05

**Authors:** Dongying Zhao, Liwei Wang, Xianting Jiao, Chutian Shi, Zhongcheng Luo, Yan Chen, Yongjun Zhang

**Affiliations:** ^1^Department of Pediatrics, Xinhua Hospital, Shanghai Jiao Tong University School of Medicine, Shanghai, China; ^2^Department of Obstetrics and Gynecology, Lunenfeld-Tanenbaum Research Institute, Mount Sinai Hospital, Faculty of Medicine, University of Toronto, Toronto, Canada; ^3^Xinhua Hospital, Shanghai Institute for Pediatric Research, Shanghai Jiao Tong University School of Medicine, Shanghai, China

**Keywords:** adolescents, hyperinsulinemia, insulin resistance, prevalence, trends

## Abstract

**Purpose:** Insulin resistance (IR)/hyperinsulinemia in young individuals is associated with the subsequent development of diabetes and cardiovascular disease. To assess trends in the prevalence of IR/hyperinsulinemia among nondiabetic/nonprediabetic adolescents in the US from 1999 to 2020.

**Methods:** A total of 6111 adolescents without diabetes and prediabetes were included from ten cycles of National Health and Nutrition Examination Survey (NHANES) between 1999–2000 and 2017–2020. Hyperinsulinemia or IR was defined as fasting insulin or homeostasis model assessment of insulin resistance [HOMA-IR] above the 75th percentile in all participants who underwent blood tests on fasting insulin, glucose, and hemoglobin A1c. Trends in prevalence rates were estimated using joinpoint regressions with heteroscedastic and uncorrelated errors.

**Results:** The overall weighted median fasting insulin level, prevalence of hyperinsulinemia, and IR were 9.9 μU/ml [95% confidence interval (CI): 9.6, 10.1], 17.2% (95% CI: 15.7, 18.6), and 16.4% (95% CI: 15.2, 17.9), respectively. The estimated prevalence of hyperinsulinemia and HOMA-IR increased significantly from 15.2% (95% CI: 12.1, 18.9) and 14.0% (95% CI: 11.1, 17.8) in 1999–2000% to 21.5% (95% CI: 17.1, 26.3) and 20.4% (95% CI: 16.4, 25.6) in 2017–2020, respectively, with a 3.35% (95% CI: 1.74, 4.99) and 3.41% (95% CI: 1.72, 5.12) relative increase per 2-year survey cycle, respectively (*p* for trend <0.05). Substantial increases were observed in the subgroups of girls, Hispanic, non-Hispanic white, and overweight adolescents.

**Conclusions:** The prevalence of hyperinsulinemia/IR increased substantially among US nondiabetic/nonprediabetic adolescents over the last two decades. Early detection and effective interventions are in dire need to reverse the rising tide.

## 1. Introduction

Diabetes is a major public health issue associated with increased mortality and disability worldwide [1]. Insulin resistance (IR)/hyperinsulinemia is recognized as a major risk factor and prelude to the development of prediabetes and type 2 diabetes [2, 3]. Population studies have shown that children with IR are much more likely to experience hypertension and elevated triglycerides in adulthood, increasing subsequent risk of atherosclerotic cardiovascular disease and type 2 diabetes [4]. Some interventions such as metformin are effective in reducing fasting insulin and IR in children and adolescents with clinical IR or prediabetes [5, 6]. Therefore, early prevention, detection, and treatment of patients with IR could help prevent the late onset of diabetes.

The prevalence of diabetes has been steadily increasing worldwide over the recent decades [1]. Among US adolescents, the overall prevalence of type 2 diabetes per 1000 adolescents increased significantly from 0.34 in 2001 to 0.67 in 2017 in six regions in the US [7]. In a previous study of National Health and Nutrition Examination Survey (NHANES) data, the prevalence of prediabetes among US youths increased significantly from 11.6% in 1999–2002 to 28.2% in 2015–2018 [8]. However, the trends in IR among nondiabetic/nonprediabetic US adolescents have not been comprehensively characterized. Knowledge of temporal trends in the prevalence of IR may inform public health policymakers and healthcare service providers in designing interventions to reverse the rising tide of diabetes. In this study, we used two decades of NHANES data (1999–2020) to characterize the trends of hyperinsulinemia/IR in adolescents without diabetes and prediabetes in the U.S.

## 2. Methods

### 2.1. Participants and Data

This study was based on the NHANES databases of the Centers for Disease Control and Prevention (CDC). The NHANES is a cross-sectional, national, representative sample survey among the U.S. civilian population of all ages. The survey data includes personal interviews, standardized physical examinations, and a series of laboratory testing, which were released every 2 years and could be obtained on the CDC website. Because of the coronavirus disease 2019 (COVID-19) pandemic, the data 2019-March 2020 had been combined with the data from 2017 to 2018 to form a nationally representative sample of NHANES 2017-March 2020 prepandemic data. Thus, we examined data on adolescents (aged 12–19 years) from 10 NHANES cycles between 1999–2000 and 2017–2020. Plasma insulin and glucose values were assessed in subjects at age 12+ years of age in the NHANES. Among adolescents, the overall response rates ranged from 54.4% to 88.5% for interviews and 50.7%–86.5% for examinations. Because the data are publicly available and anonymized, institutional review board approval was not required for this study.

### 2.2. Insulin Measurements and Definitions of Hyperinsulinemia and Insulin Resistance

Blood samples were collected on scheduled morning participants who fasted for 8 to less than 24 h to assess plasma insulin and glucose. Assay methods are available in the CDC NHANES website (https://wwwn.cdc.gov/nchs/nhanes/default.aspx). As the laboratory method and equipment have changed over time, fasting insulin and glucose were calibrated to the earlier NHANES surveys using backward equations to the 2003–2004 cycle as recommended by the National Center for Health Statistics. However, hemoglobin A1c (HbA1c) values do not need to be calibrated according to NHANES [9].

The focus of this study was on insulin status in adolescents without diabetes and prediabetes. Hyperinsulinemia and IR were the primary outcomes. Adolescents aged 12–19 years who underwent lab tests for fasting insulin, glucose, and HbA1c in NHANES cycles during 1999–2020 were included. Hyperinsulinemia was defined as fasting insulin above the 75th percentile cutoff in the aforementioned participants. Homeostasis model assessment (HOMA) was used to evaluate IR: HOMA-IR = [glucose (millimoles per liter) × insulin (microunits per milliliter)]/22.5). HOMA-IR above the 75th percentile cutoff in the same participants was considered to have IR [10]. The cutoff values of fasting insulin concentration and HOMA-IR were 17.33 μU/ml and 3.96, respectively. The study population excluded participants with diabetes or prediabetes. Diabetes was defined as (1) self-reports of diabetes diagnosis by a physician or other health professional, or currently taking diabetic pills or insulin; or (2) having a fasting plasma glucose level equal to or above 126 mg/dL or HbA1c level equal or above 6.5%, as defined in previous studies [11]. Prediabetes was defined as a fasting plasma glucose level of 100–125 mg/dL or an HbA1c level of 5.7%–6.4% among the participants without a diagnosis of diabetes [8]. A final total sample included 6111 adolescents.

In the NHANES, information on age, sex, race/ethnicity, and poverty income ratio (PIR) was collected during household interviews. Race/ethnicity was categorized as Hispanic (Mexican American and other Hispanic), non-Hispanic white, non-Hispanic black, and other races. PIR was defined as low and high (PIR < 1.85 and ≥ 1.85) according to previous studies [12]. Body mass index (BMI) was classified into underweight, normal weight, overweight, and obese according to the WHO Standard of Adolescents.

### 2.3. Statistical Analysis

For demographic characteristics, results are expressed as weighted mean ± standard deviation for continuous variables, unweighted count, and percentage for categorical variables. For insulin and related parameters, median or prevalence estimates with 95% confidence intervals (CIs) are presented.

Trends in prevalence rates of hyperinsulinemia and IR were assessed via joinpoint regressions with heteroscedastic and uncorrelated errors. Relative percentage change per 2-year NHANES cycle in median or prevalence with 95% CIs was calculated. Mixed effects models were introduced to assess the differences in the insulin levels and the prevalence of IR/hyperinsulinemia over time among subgroups. Weights for the interview sample and fasting subsample were used to approximate the total civilian noninstitutionalized US adolescents. Two-tailed *p* values < 0.05 indicate statistical significance. Statistical analysis was performed using SAS version 9.2 (SAS Institute Inc., Cary, North Carolina) and Joinpoint Regression Program version 4.9.0.0.

## 3. Results

### 3.1. Characteristics of Participants

In the final study sample including 6111 participants aged 12–19 years, there were 347–885 subjects per 2-year cycle. The weighted mean age was 15.52 ± 0.04. Males accounted for 48.4% of study subjects. There were 60.1% non-Hispanic white, 13.6% non-Hispanic black, and 18.7% Hispanic American (weighted proportions). The mean age and proportions of BMI categories remained stable across the ten NHANES cycles. There has been a shift in the ethnic distribution over the ten cycles, with a significant increase in the proportion of Hispanic and other races (Table 1).

### 3.2. Trends of Insulin-Related Parameters

The overall weighted median fasting insulin level, prevalence rates of hyperinsulinemia, and IR were 9.9 μU/ml (95% CI: 9.6, 10.1), 17.2% (95% CI: 15.7, 18.6), and 16.4% (95% CI: 15.2, 17.9), respectively.

**Figure 1:**
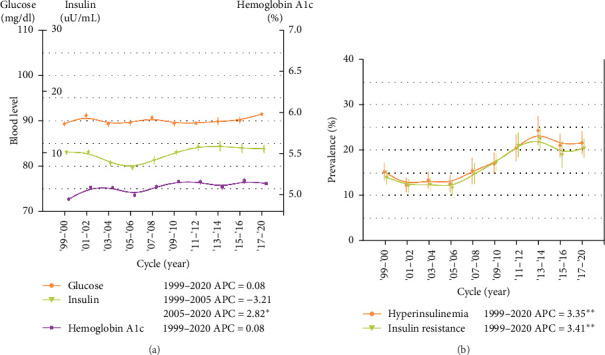
Trends in plasma insulin, glucose, hemoglobin A1c, and prevalence rates of hyperinsulinemia and insulin resistance among nondiabetic/nonprediabetic adolescents in the US, 1999–2020. The error bars represented the weighted median insulin, glucose, and hemoglobin A1c (A) and prevalence rates of hyperinsulinemia and insulin resistance (B) with 95% confidence intervals. *P* values for trend and annual percent change (APC) were obtained from joinpoint regressions. *p* < 0.05 for insulin in (A), *p* < 0.01 for the prevalence rates of hyperinsulinemia and insulin resistance in (B). There were no temporal trends in fasting glucose and hemoglobin A1c levels. Specific estimates are presented in Table 2 and Supporting Information Table S1. *⁣*^*∗*^, *p* < 0.05, *⁣*^*∗∗*^, *p* < 0.01.

Plasma insulin levels trended higher between 2005–2006 and 2017–2020, with a 2.82 μU/ml (95% CI: 0.38, 5.33) relative increase per 2-year NHANES cycle (*p* < 0.05 for trend). Consistently, the prevalence rates of hyperinsulinemia and IR increased substantially from 15.2% (95% CI: 12.1, 18.9) and 14.0% (95% CI: 11.1, 17.8) in 1999–2000 to 21.5% (95% CI: 17.1–26.3) and 20.4% (95% CI: 16.4–25.6) in 2017–2020, respectively, with a 3.35% (95% CI: 1.74, 4.99, *p*=0.001) and 3.41% (95% CI: 1.72, 5.12, *p*=0.002) relative increase per 2-year NHANES cycle, respectively. Population-attributable analyses indicated that 35.6% (hyperinsulinemia) and 38.5% (IR) of these increases can be attributable to the Hispanic population growth, respectively. No significant increases in fasting blood glucose and HbA1c levels were observed over the ten NHANES cycle (*p* > 0.05, all) (Figure 1, Table 2, Supporting Information Table S1).

**Figure 2:**
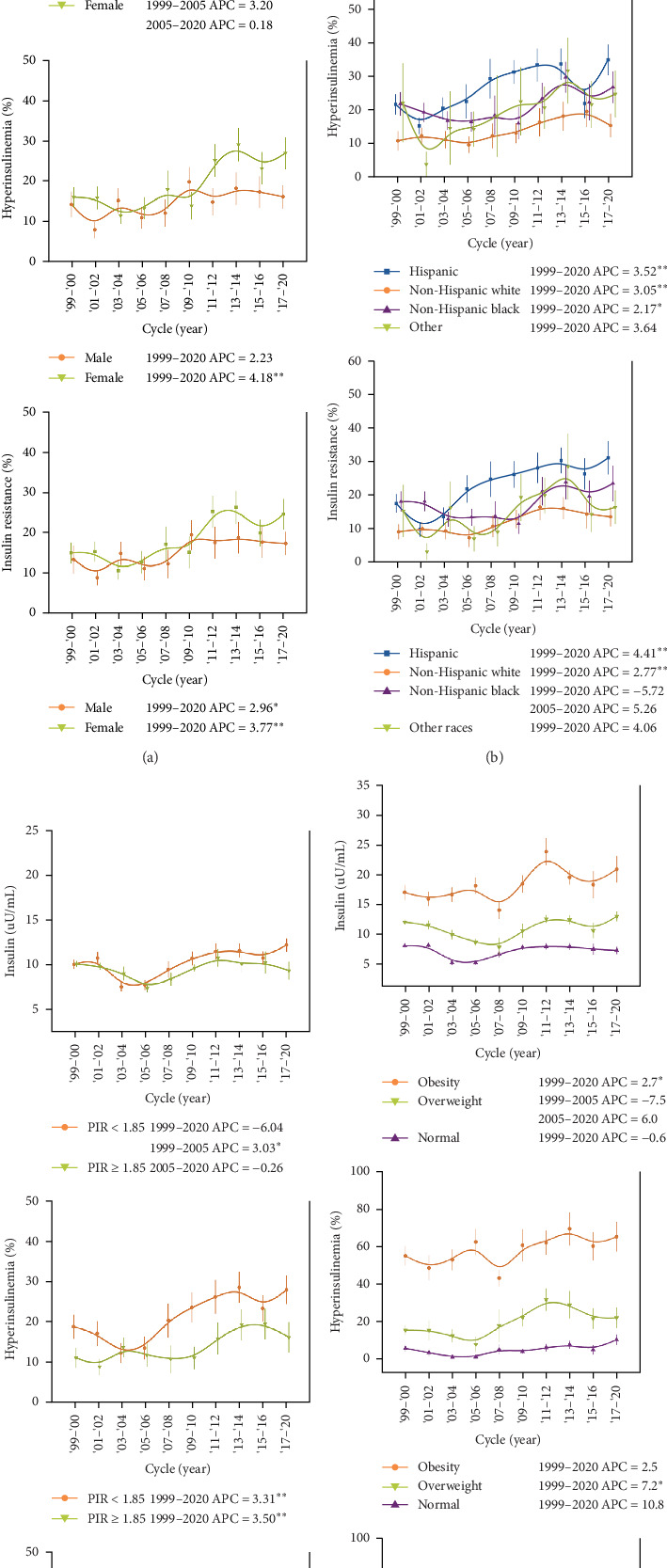
Trends in insulin levels and prevalence rates of hyperinsulinemia and insulin resistance among sociodemographic subgroups in the US, 1999–2020. Trends in insulin levels and prevalence rates of hyperinsulinemia and insulin resistance stratified by sex (A), race/ethnicity (B), poverty index ratio (C), and body mass index (D). Error bars indicate 95% CIs. *P* values for trends and annual percent change (APC) were obtained from joinpoint regressions. Significant upward trends in hyperinsulinemia and insulin resistance were observed in the subgroups of girls, Hispanic, non-Hispanic white, both group of PIR, and overweight groups. Specific estimates are presented in Table 2. PIR, poverty income ratio. *⁣*^*∗*^, *p* < 0.05, *⁣*^*∗∗*^, *p* < 0.01.

In stratified analyses, for fasting insulin levels, apparent upward trends could only be observed in a few hierarchies after 2003–2006, i.e., in Hispanic individuals, other racial groups, participants with a PIR < 1.85 and obesity. The prevalence of hyperinsulinemia and HOMA-IR was confirmed to trend higher in most hierarchies, except for males, non-Hispanic blacks, other races, those with normal weight, and those with obesity. Girls seemed to have greater relative changes in the prevalence of hyperinsulinemia (4.18% vs. 2.23%) and HOMA-IR (3.77% vs. 2.96%). Hispanic Americans had a higher prevalence in both hyperinsulinemia and HOMA-IR than non-Hispanic white and other races (Table 3). Increasing trends of hyperinsulinemia and HOMA-IR were mainly observed in Hispanic [annual percent change (APC): 3.52 (95% CI: 91.13, 5.97) and 4.41 (95% CI: 2.02, 6.86)] and non-Hispanic white adolescents [APC: 3.05 (95% CI: 1.26, 4.87) and 2.77 (95% CI: 1.10, 4.47)] (Table 2, Figure 2). The prevalence of hyperinsulinemia and HOMA-IR was higher in those with PIR < 1.85 (Table 3), while the rising trends were observed in both PIR groups, with APC = 3.31 (95% CI: 1.11, 5.56) and 3.68 (95% CI: 1.48, 5.92) for PIR < 1.85, APC = 3.50 (95% CI: 1.38, 5.65) and 3.08 (95% CI: 0.61, 5.61) for PIR ≥ 1.85, respectively (Figure 2, Table 2). Notably, participants in the low PIR group demonstrated a significantly higher obesity prevalence compared to their high PIR counterparts (21.0% vs. 17.5%, *p*=0.001). While both obese and overweight groups exhibited a higher prevalence of hyperinsulinemia and HOMA-IR compared to the normal-weight group, a significant upward trend was observed exclusively in the overweight cohort. In the sensitivity analyses, when participants were restricted to those with normal HbA1c, the upward trends remained in girls, Hispanic Americans, other races, as well as adolescents with a PIR < 1.85 (all *p* < 0.05) (Supporting Information Table S1, Figure S1). There were racial differences in fasting glucose and HbA1c levels (Supporting Information Table S1, Figure S1, Table S2).

### 3.3. Discussion

The study results indicate that insulin levels as well as the prevalence rates of hyperinsulinemia and IR are on the rise among adolescents without diabetes/prediabetes over the last two decades. The median fasting insulin concentrations increased by 2.82 μU/ml per 2-year NHANES cycle, while the prevalence rates of hyperinsulinemia and IR increased by 3.35% and 3.41% (relative increase) per 2-year NHANES cycle, respectively.

Previous studies have largely focused on diabetes and its precursor prediabetes [1, 7, 8, 11, 13]. However, it is now recognized that before abnormal glucose levels are detectable, a status of IR is a common presentation. As this status continues, blood glucose levels would rise to the point of diagnosis of prediabetes or diabetes [2, 14]. Besides, IR has been associated with metabolic syndrome, cardiovascular disease, metabolic-associated fatty liver disease, polycystic ovary syndrome, and certain cancers [14]. Thus, early detection and effective treatment of hyperinsulinemia/IR especially in conditions of euglycemia in young individuals may help prevent type 2 diabetes/prediabetes as well as other diseases in adulthood.

It has been demonstrated that fasting insulin and the prevalence of hyperinsulinemia have increased remarkably among nondiabetic US adults [15]. Consistent with this observation, in this study, we demonstrated that both the prevalence of hyperinsulinemia and IR in nondiabetic/nonprediabetic adolescents have been increasing at a rate of more than 3% every 2 years during the last two decades in the US. IR is one of the pathophysiological alterations of type 2 diabetes. The transition from normal glucose tolerance to impaired glucose tolerance to type 2 diabetes in youth may often be accompanied by progressive increases in IR [16]. The increased HOMA-IR during puberty could induce a worsening of endothelial function even at a young age, and increase cardiovascular risk [17]. Therefore, more efforts should be undertaken to optimize IR during adolescence.

Our study also identified significant subgroup disparities in IR metrics across race/ethnicity, sex, poverty status, and BMI categories. Building upon previous findings by Moran et al. [18], which described a sexually dimorphic pattern of IR favoring adolescent females, our findings corroborated that females exhibited greater relative increases in both hyperinsulinemia and HOMA-IR prevalence over time compared to males. This disparity may be mechanistically linked to sex-specific adiposity distribution, as females tend to accumulate subcutaneous adipose tissue preferentially in regions such as the triceps and subscapular areas—sites strongly associated with obesity-related IR [18, 19]. These results highlight the necessity for gender-targeted prevention strategies. The ethnically stratified analysis revealed that Hispanic Americans had a higher prevalence of IR than other non-Hispanic races. Approximately one-third of the observed increases in hyperinsulinemia and IR can be attributed to growth in the Hispanic population. The underlying mechanisms may be attributed to the capacity for insulin clearance which was negatively associated with Hispanic status [20, 21]. However, it is worth noting that non-Hispanic white had almost the same increase as the Hispanic in the prevalence of hyperinsulinemia (3.05% and 3.52%), even as the former had a low prevalence of hyperinsulinemia. Childhood poverty has been demonstrated as a contributor to IR in adulthood [22]. In this study, the prevalence of IR was confirmed to be higher among adolescents with PIR < 1.85 (vs. PIR ≥ 1.85). This subgroup also exhibited higher obesity prevalence. Mechanistically, obesity, biological (i.e., immune cell aging), or psychological (i.e., perceived stress) may partly account for the differences [22, 23]. Notably, despite stable population-level BMI distributions over time, overweight adolescents demonstrated a significant progressive increase in both hyperinsulinemia rate and HOMA-IR levels during the study period. These findings underscore the central role of BMI as the primary predictor of IR risk [24]. Among overweight individuals—even those not meeting obesity criteria—multidomain lifestyle interventions targeting structured dietary control, increased physical activity, and circadian rhythm optimization are warranted to enhance insulin sensitivity and mitigate disease progression.

A strength of this study was the use of 20 years of a large nationally representative study sample and high-quality data. Our study had limitations. First, not all adolescents underwent blood tests on fasting insulin, glucose, and HbA1c. Second, besides fasting insulin level and HOMA-IR, other methods such as hyperinsulinemic–euglycemic clamp, which could assess IR more precisely [25], were not available in the NHANES.

In conclusion, NHANES data on nondiabetic/nonprediabetic adolescents revealed substantial increases in fasting insulin and the prevalence of hyperinsulinemia and IR over the last two decades in the US. This represents a major public health concern as those affected have no apparent clinical symptoms—a hidden iceberg before the advent of diabetes/prediabetes. There is a dire need for optimal strategies and interventions to reverse the rising tide.

## Figures and Tables

**Table 1:** Characteristics of nondiabetic/nonprediabetic adolescents in the US by survey cycles according to NHANES 1999–2020.

Characteristic	1999–2000	2001–2002	2003–2004	2005–2006	2007–2008	2009–2010	2011–2012	2013–2014	2015–2016	2017–2020	P trends
*n*, %	859 (9.1)	856 (11.1)	885 (10.3)	775 (10.3)	347 (9.5)	501 (10.4)	425 (10.0)	483 (9.9)	407 (9.8)	573 (9.6)	—
Age (years)	15.4 (0.1)	15.5 (0.1)	15.6 (0.2)	15.6 (0.1)	15.8 (0.1)	15.3 (0.1)	15.3 (0.2)	15.6 (0.1)	15.5 (0.2)	15.5 (0.1)	0.868
Sex (*n*, %)											
Male	424 (51.5)	385 (46.3)	456 (48.7)	360 (46.8)	184 (47.5)	259 (49.1)	197 (48.9)	220 (46.0)	199 (48.7)	285 (50.8)	**0.008**
Female	435 (48.5)	471 (53.7)	429 (51.3)	415 (53.2)	163 (52.5)	242 (50.9)	228 (51.1)	263 (54.0)	208 (51.3)	288 (49.2)	**0.006**
Race (*n*, %)											
Hispanic	386 (19.0)	261 (13.7)	274 (16.9)	269 (15.1)	119 (16.4)	187 (18.2)	100 (19.5)	156 (22.2)	118 (21.4)	149 (25.3)	**0.001**
Non-Hispanic white	182 (59.3)	249 (64.2)	211 (62.8)	188 (63.4)	115 (65.2)	162 (61.2)	112 (59.5)	115 (55.4)	105 (55.8)	178 (53.1)	0.067
Non-Hispanic black	212 (14.3)	237 (13.5)	306 (15.2)	237 (14.4)	72 (13.4)	83 (13.0)	119 (13.5)	102 (14.5)	84 (12.2)	106 (11.3)	**0.012**
Other races	29 (7.4)	40 (8.6)	33 (5.1)	35 (7.1)	14 (5.0)	34 (7.6)	72 (7.5)	64 (7.9)	63 (10.6)	91 (10.3)	**0.003**
Poverty income ratio (*n*, %)											
Below poverty level (<1.85)	451 (45.2)	410 (36.2)	500 (47.5)	400 (36.3)	178 (38.5)	255 (38.3)	227 (46.1)	271 (47.2)	213 (38.6)	280 (41.9)	**0.008**
At or above poverty level (≥1.85)	301 (54.8)	398 (63.8)	335 (52.5)	338 (63.7)	143 (61.5)	199 (61.7)	163 (53.9)	179 (52.8)	154 (61.4)	233 (58.1)	**0.014**
Body mass index (*n*, %)											
Underweight	20 (4.2)	23 (3.0)	21 (2.4)	23 (4.1)	7 (1.6)	8 (1.4)	14 (2.0)	13 (2.6)	5 (1.7)	13 (2.7)	0.148
Normal weight	507 (63.5)	549 (68.1)	533 (62.0)	465 (65.4)	201 (60.7)	296 (62.7)	264 (65.8)	284 (62)	234 (54.5)	320 (62.1)	0.051
Overweight	157 (16.0)	137 (13.1)	152 (16.6)	121 (14.0)	60 (18.2)	106 (19.7)	60 (12.2)	77 (13.4)	83 (21.9)	115 (17.4)	0.166
Obese	172 (16.3)	134 (15.7)	168 (19.1)	165 (16.5)	70 (19.5)	82 (16.1)	78 (20.0)	102 (22.0)	83 (21.8)	115 (17.7)	0.061

*Note:* Data presented are weighted means, unweighted counts, and weighted percentage. Bold values stand for *p* < 0.05.

**Table 2:** Trends in insulin related parameters among nondiabetic/nonprediabetic adolescents in the US, 1999–2020.

Characteristic	1999–2000	2001–2002	2003–2004	2005–2006	2007–2008	2009–2010	2011–2012	2013–2014	2015–2016	2017–2020	Relative change per 2-y cycle
	Median (95% CI)	Median (95% CI)	Median (95% CI)	Median (95% CI)	Median (95% CI)	Median (95% CI)	Median (95% CI)	Median (95% CI)	Median (95% CI)	Median (95% CI)	(95% CI)
Fasting insulin (μU/ml)											
Overall	10.1 (9.6, 10.7)	10.1 (9.5, 10.8)	8.4 (7.6, 9.1)	7.6 (6.9, 8.3)	8.8 (7.6, 10.0)	10.1 (9.4, 10.8)	11.0 (9.9, 12.1)	11.1 (9.6, 12.6)	10.8 (9.5, 12.1)	10.7 (9.5, 11.8)	1999–2005: −3.21 (−7.67, 1.47)
											2005–2020: 2.82 (0.38, 5.33)*⁣*^*∗*^
Sex											
Male	9.4 (8.5, 10.3)	9.4 (8.9, 10.0)	7.2 (6.4, 8.1)	6.7 (6.0, 7.4)	8.3 (7.4, 9.2)	10.6 (9.3, 11.9)	9.7 (8.3, 11.0)	9.8 (7.8, 11.7)	9.1 (7.2, 11.1)	9.9 (8.2, 11.5)	−5.69 (−12.08, 1.18)
Female	11.0 (10.3, 11.8)	10.7 (9.9, 11.5)	9.2 (8.1, 10.4)	8.6 (7.5, 9.6)	9.3 (7.9, 10.7)	10.1 (8.7, 11.4)	12.9 (11.6, 14.3)	11.6 (9.8, 13.5)	11.7 (10, 13.5)	11.4 (9.0, 13.9)	1999–2005: 3.20 (1.5, 4.93)
											2005–2020: 0.18 (−1.31, 1.71)
Race											
Hispanic	11.1 (10.4, 11.8)	10.3 (8.7, 11.9)	8.1 (6.9, 9.2)	9.3 (7.7, 10.9)	9.5 (8.1, 10.8)	12.4 (10.8, 13.9)	11.7 (9.8, 13.6)	12.6 (11.2, 14.1)	12.1 (9.9, 14.3)	12.2 (10.7, 13.7)	1999–2003: −5.31 (−17.99, 9.34)
											2003–2020: 2.91 (0.50, 5.38)*⁣*^*∗*^
Non-Hispanic white	9.6 (8.9, 10.4)	10.0 (9.1, 11.0)	8.4 (7.4, 9.5)	7.2 (6.2, 8.2)	8.6 (7.5, 9.8)	10 (9.2, 10.8)	10.7 (8.7, 12.7)	10.2 (8.0, 12.4)	9.7 (8.0, 11.4)	9.6 (7.3, 11.9)	0.18 (−1.31, 1.71)
Non-Hispanic black	11.3 (10.3, 12.3)	10.8 (10, 11.7)	8.3 (7.4, 9.2)	8.1 (6.6, 9.7)	10.6 (8.3, 12.9)	10.1 (8.7, 11.4)	11.8 (9.7, 13.9)	12.0 (9.1, 14.9)	11.0 (10.1, 11.8)	11.9 (10.5, 13.4)	1999–2003: −6.95 (−14.34, 1.08)
											2003–2020: 2.12 (0.21, 4.06)*⁣*^*∗*^
Other races	11.3 (8.6, 13.9)	9.7 (8.6, 10.8)	7.6 (4.1, 11.2)	7.2 (4.5, 9.9)	6.7 (2.4, 11.0)	8.9 (4.6, 13.2)	10.3 (6.4, 14.3)	11.0 (6.4, 15.5)	9.9 (7.6, 12.3)	10.6 (8.7, 12.4)	1999–2005: −6.56 (−13.13, 0.5)
											2005–2020: 3.21 (0.39, 6.12)*⁣*^*∗*^
Poverty income ratio											
Below poverty level (<1.85)	10.1 (9.2, 11.0)	10.8 (9.5, 12.1)	7.7 (6.7, 8.6)	7.8 (6.8, 8.8)	9.6 (7.9, 11.3)	10.8 (9.3, 12.2)	11.5 (9.9, 13.2)	11.7 (10.2, 13.1)	10.8 (9.5, 12.2)	12.2 (10.9, 13.6)	1999–2003: −6.04 (−17.52, 7.04)
											2003–2020: 3.03 (0.87, 5.23)*⁣*^*∗*^
At or above poverty level (≥1.85)	10.2 (9.6, 10.8)	9.9 (9.2, 10.6)	9.1 (7.6, 10.6)	7.5 (6.7, 8.3)	8.5 (7.2, 9.8)	9.7 (9.1, 10.4)	10.8 (9.0, 12.7)	10.1 (8.3, 12.0)	10.3 (8.0, 12.7)	9.4 (7.5, 11.3)	−0.26 (−1.68, 1.18)
Body mass index (*n*, %)											
Underweight	7.9 (4.5, 11.4)	7.1 (4.9, 9.3)	—	5.1 (3.1, 7.1)	—	7.9 (5.5, 10.4)	6.5 (4.4, 8.6)	9.1 (6.7, 11.4)	—	9.1 (5.7, 12.6)	—
Normal weight	9.2 (8.6, 9.7)	9.2 (8.6, 9.8)	6.4 (5.7, 7.2)	6.4 (5.9, 7.0)	7.8 (6.9, 8.6)	8.9 (8.1, 9.7)	9.1 (8.1, 10)	9.0 (8.0, 10.1)	8.6 (6.9, 10.3)	8.4 (7.3, 9.5)	−0.6 (−4.4, 3.5)
Overweight	12.9 (12.2, 13.7)	12.5 (11.1, 13.9)	10.9 (9.4, 12.4)	9.7 (8.7, 10.8)	9.0 (6.1, 11.8)	11.6 (9.3, 13.8)	13.5 (12.2, 14.7)	13.3 (11.9, 14.8)	11.6 (9.2, 14.0)	13.9 (12.3, 15.4)	1999–2005: −7.5 (−17.3, 3.3)
											2005–2020: 6 (−0.6, 13)
Obese	17.7 (15.3, 20.1)	16.7 (14.5, 18.9)	17.4 (15.1, 19.6)	18.8 (16.2, 21.3)	14.9 (12.1, 17.7)	19.1 (16.4, 21.7)	24.2 (19.9, 28.6)	20.1 (17.9, 22.4)	19 (14.7, 23.2)	21.4 (17.2, 25.6)	2.7 (0.2, 5.2)*⁣*^*∗*^
Hyperinsulinemia (%)											
Overall	15.2 (12.1, 18.9)	12.3 (9.4, 15.3)	13.4 (10.4, 16.9)	12.5 (9.1, 16.2)	15.4 (11.2, 20.9)	17 (13.3, 21.5)	20.4 (15.6, 25.9)	24.2 (19.4, 30.2)	20.9 (15.2, 25.8)	21.5 (17.1, 26.3)	3.35 (1.74, 4.99)*⁣*^*∗∗*^
Sex											
Male	14.5 (10.2, 20.5)	8.2 (4.7, 12.0)	15.4 (10.0, 21.3)	11.2 (6.7, 16.3)	12.3 (7.6, 18.8)	20.0 (14, 27.1)	15.1 (9.5, 21.5)	18.3 (11.5, 26.0)	17.5 (10.8, 25.0)	16.3 (10.8, 21.7)	2.23 (−0.50, 5.03)
Female	16.1 (11.8, 21.0)	16.0 (11.2, 21.3)	11.7 (8.0, 15.4)	13.5 (8.8, 18.6)	18.1 (10.7, 27.1)	14.1 (9.2, 20.6)	25.2 (17.4, 32.9)	29.1 (22.1, 37.1)	23.3 (16.5, 30.9)	27.0 (20.8, 34.5)	4.18 (1.67, 6.76)*⁣*^*∗∗*^
Race											
Hispanic	21.4 (15.6, 27.2)	11.3 (7.3, 15.9)	17.6 (11.5, 23.8)	23.8 (16.5, 33.6)	26.7 (17.2, 38.0)	29.2 (22.1, 35.9)	29.1 (20.7, 38.5)	34.3 (26.8, 43.2)	25.6 (16.7, 33.9)	33.8 (24.5, 42.6)	3.52 (1.13, 5.97)*⁣*^*∗∗*^
Non-Hispanic white	10.8 (6.2, 16.4)	12.3 (8.1, 17.0)	11.2 (7.0, 16.0)	9.7 (4.3, 14.3)	12.3 (6.2, 19.3)	13.1 (8.0, 18.7)	16.4 (9.3, 24.3)	18.1 (11.8, 26.3)	19.5 (11.0, 28.1)	15.4 (10.0, 22.0)	3.05 (1.26, 4.87)*⁣*^*∗∗*^
Non-Hispanic black	21.8 (16.0, 28.5)	19.3 (14.4, 24.7)	16.8 (12.2, 22.0)	16.5 (11.1, 22.0)	18.4 (9.9, 29.7)	16.1 (8.9, 25.3)	23.4 (15.4, 32.2)	29.6 (19.9, 38.4)	22.3 (13.7, 32.6)	26.7 (16.8, 35.8)	2.17 (0.08, 4.30)*⁣*^*∗*^
Other races	23.2 (5.3, 46.0)	3.6 (0.4, 10.9)	17.7 (3.0, 38.7)	7.6 (1.6, 17.6)	10.7 (2.8, 34.2)	20.6 (7.4, 40.2)	20.1 (10.2, 32.3)	27.7 (10.7, 46.6)	14.7 (5.0, 28.1)	18.2 (9.2, 31.7)	3.64 (−0.54, 7.99)
Poverty income ratio											
Below poverty level (<1.85)	18.9 (13.2, 24.6)	17.3 (12.1, 23.1)	12.5 (8.9, 17.2)	13.7 (9.2, 19.2)	20.5 (13.9, 28.5)	23.7 (17.6, 30.8)	26.3 (18.8, 34.3)	28.6 (20.9, 35.9)	23.4 (16.7, 29.7)	28.0 (21.2, 34.7)	3.31 (1.11, 5.56)*⁣*^*∗∗*^
At or above poverty level (≥1.85)	11.3 (7.1, 16.0)	9.1 (5.6, 12.8)	13.9 (9.3, 18.6)	11.9 (7, 17.4)	10.9 (5.5, 17.5)	11.3 (6.4, 16.6)	16.0 (8.3, 23.5)	19.4 (11.4, 26.7)	19.6 (10.8, 26.8)	16.3 (10.5, 23.5)	3.50 (1.38, 5.65)*⁣*^*∗∗*^
Body mass index (*n*, %)											
Underweight	—	—	—	—	—	—	—	—	—	—	—
Normal weight	6.6 (2.8, 10.3)	4.3 (1.1, 7.5)	2.1 (0.4, 3.9)	2.1 (0.7, 3.6)	6.0 (0.5, 11.4)	4.9 (2.6, 7.2)	6.8 (3.1, 10.6)	8.4 (4.3, 12.5)	5.8 (0.6, 11)	11.0 (5.8, 16.1)	10.8 (−0.5, 23.3)
Overweight	15.8 (10.5, 21.1)	15.7 (5.0, 26.5)	13 (6.1, 19.8)	8.6 (0.5, 16.6)	18.1 (0.8, 35.4)	22.4 (12.8, 31.9)	31.4 (19.5, 43.3)	28.8 (14.1, 43.5)	21.7 (10.6, 32.9)	22.1 (11.4, 32.8)	7.2 (1, 13.9)*⁣*^*∗*^
Obese	53.7 (43.4, 64.1)	47.6 (33.9, 61.2)	51.7 (40.7, 62.8)	60.9 (46.6, 75.1)	42.4 (33.3, 51.4)	59.1 (41.7, 76.5)	60.5 (47.8, 73.2)	67.5 (49.5, 85.5)	58.7 (43.7, 73.8)	63.5 (47.9, 79.1)	2.5 (−0.7, 5.9)
HOMA-IR (%)											
Overall	14.0 (11.1, 17.8)	12.1 (9.3, 15.3)	12.5 (9.4, 15.6)	11.7 (8.5, 15.6)	14.8 (10.5, 20.4)	17.3 (13.4, 22.2)	21.1 (16.0, 26.1)	22.5 (17.9, 28.5)	18.9 (13.5, 23.3)	20.4 (16.4, 25.6)	3.41 (1.72, 5.12)*⁣*^*∗∗*^
Sex											
Male	13.2 (9.1, 19.7)	8.7 (5.0, 12.4)	14.7 (9.4, 20.2)	11.0 (6.4, 16.6)	12.1 (7.3, 18.8)	19.3 (13.5, 26.4)	17.3 (10.4, 24.9)	18.6 (11.4, 26.1)	17.5 (10.8, 25.0)	17.1 (11.3, 22.7)	2.96 (0.57, 5.42)*⁣*^*∗*^
Female	14.8 (10.3, 19.7)	15.1 (10.5, 19.9)	10.5 (7.0, 14.7)	12.5 (8.0, 17.8)	16.9 (10.0, 25.5)	14.9 (9.6, 22.5)	24.9 (17.3, 32.6)	26.0 (19.5, 33.9)	19.7 (13.4, 26.1)	24.3 (18.6, 31.6)	3.77 (1.31, 6.29)*⁣*^*∗∗*^
Race											
Hispanic	18.9 (13.9, 24.9)	11.5 (7.4, 16.1)	15.1 (10.1, 21.2)	23.0 (15.5, 32.5)	25.8 (16.0, 36.5)	27.2 (19.8, 33.8)	29.1 (20.7, 38.5)	31.2 (24.1, 38.9)	27.3 (18.6, 36.3)	32.0 (22.7, 40.3)	4.41 (2.02, 6.86)*⁣*^*∗∗*^
Non-Hispanic white	10.7 (6.6, 16.6)	11.6 (7.7, 16.4)	11.0 (6.4, 15.8)	9.0 (4.1, 13.8)	12.3 (6.2, 19.3)	14.4 (9.4, 21.4)	17.8 (10.6, 26.9)	17.5 (11.4, 25.7)	15.8 (7.9, 22.4)	15.1 (9.8, 22.0)	2.77 (1.10, 4.47)*⁣*^*∗∗*^
Non-Hispanic black	19.4 (13.9, 26.0)	19.5 (14.1, 24.0)	14.4 (10.4, 18.5)	14.9 (10.3, 19.4)	15.3 (7.0, 25.5)	13.0 (7.5, 21.7)	22.4 (14.6, 32.2)	25.0 (15.7, 32.5)	20.9 (12.0, 30.6)	24.6 (14.7, 33.4)	1999–2005: −5.72 (−16.48, 6.43)
											2005–2020: 5.26 (−1.35, 12.31)
Other races	16.7 (2.2, 38.0)	5.1 (0.5, 15.0)	17.7 (3.0, 38.7)	8.7 (1.8, 19.3)	10.7 (2.8, 34.2)	20.6 (7.4, 40.2)	21.2 (11.3, 32.5)	29.4 (11.1, 46.6)	15.8 (5.9, 29.2)	17.8 (8.5, 28.9)	4.06 (−1.50, 9.93)
Poverty income ratio											
Below poverty level (<1.85)	16.4 (11.2, 21.7)	17.6 (12.7, 24.2)	9.9 (6.9, 14.7)	13.8 (9.6, 20.4)	20.2 (13.1, 28.1)	23.5 (17.2, 30.7)	26.4 (18.7, 34.3)	26.9 (19.5, 34.5)	24.3 (17.3, 30.9)	27.4 (21.0, 33.8)	3.68 (1.48, 5.92)*⁣*^*∗∗*^
At or above poverty level (≥1.85)	11.0 (7.0, 15.7)	8.6 (5.4, 12.0)	14.6 (9.7, 19.4)	10.6 (6.4, 15.0)	10.1 (5.0, 16.8)	11.8 (7.3, 18.4)	17.6 (9.7, 25.9)	18.5 (10.5, 26.2)	15.5 (8.1, 22.4)	16.2 (11.0, 23.5)	3.08 (0.61, 5.61)*⁣*^*∗*^
Body mass index (*n*, %)											
Underweight	—	—	—	—	—	—	—	—	—	—	—
Normal weight	5.9 (2.0, 9.9)	4.1 (1.4, 6.8)	1.9 (0.3, 3.5)	2.7 (1.0, 4.3)	5.9 (0.9, 11.0)	4.2 (1.9, 6.6)	7.7 (2, 13.4)	7.7 (4.1, 11.2)	3.6 (0.7, 6.6)	10.3 (5.6, 15.1)	10.9 (−0.2, 23.4)
Overweight	15.0 (9.8, 20.2)	14.9 (3.9, 26.0)	11.9 (5.5, 18.4)	7.8 (0, 15.9)	16.1 (0, 33.1)	21.4 (13.1, 29.6)	32.1 (20.4, 43.8)	25.1 (8.3, 42.0)	21.7 (10.6, 32.9)	20.9 (10.4, 31.3)	7.5 (0.6, 14.9)*⁣*^*∗*^
Obese	49.7 (39.8, 59.6)	47.0 (31.6, 62.4)	48.7 (39.3, 58.2)	55.4 (42.9, 67.9)	41.3 (32.5, 50.0)	63.1 (46.7, 79.5)	60.6 (47.6, 73.7)	64.2 (46.0, 82.5)	55.5 (39.7, 71.3)	61.4 (46.0, 76.9)	3.1 (−0.2, 6.5)

*Note:* All estimates are weighted.

Abbreviation: HOMA-IR, homeostasis model assessment-insulin resistance.

*⁣* ^*∗*^ *p* < 0.05, *⁣*^*∗∗*^*p* < 0.01.

**Table 3:** Differences in insulin related parameters among subgroups (regression models).

Characteristics	Crude *β* (95% CI)	*p* Value	Adjusted *β* (95% CI)^a^	*p* Value
Insulin (μU/ml)				
Race				
Hispanic	Ref	Ref	Ref	Ref
Non-Hispanic white	−2.5 (−3.19, −1.82)	**<0.0001**	−1.58 (−2.2, −0.97)	**<0.0001**
Non-Hispanic black	−0.53 (−1.22, 0.16)	0.13	−0.39 (−0.99, 0.21)	0.199
Other races	−1.68 (−2.74, −0.63)	**0.002**	−0.48 (−1.4, 0.45)	0.313
Sex				
Male	Ref	Ref	Ref	Ref
Female	2.14 (1.6, 2.68)	**<0.0001**	−0.57 (−1.06, −0.08)	**0.024**
Poverty income ratio				
Below poverty level (<1.85)	Ref	Ref	Ref	Ref
At or above poverty level (≥1.85)	−1.44 (−1.98, −0.89)	**<0.0001**	1.81 (1.34, 2.28)	**<0.0001**
Body mass index (*n*, %)				
Underweight	−2.26 (−3.79, −0.73)	**0.004**	−2 (−3.51, −0.48)	**0.010**
Normal weight	Ref	Ref	Ref	Ref
Overweight	4 (3.36, 4.63)	**<0.0001**	3.83 (3.2, 4.47)	**<0.0001**
Obese	12.97 (12.35, 13.58)	**<0.0001**	12.8 (12.19, 13.41)	**<0.0001**
Hyperinsulinemia (%)				
Race				
Hispanic	Ref	Ref	Ref	Ref
Non-Hispanic white	−0.1 (−0.13, −0.08)	**<0.0001**	−0.07 (−0.09, −0.05)	**<0.0001**
Non-Hispanic black	−0.03 (−0.06, 0)	**0.026**	−0.03 (−0.05, 0)	**0.023**
Other races	−0.07 (−0.11, −0.03)	**0.001**	−0.03 (−0.06, 0.01)	0.168
Sex				
Male	Ref	Ref	Ref	Ref
Female	0.07 (0.05, 0.09)	**<0.0001**	−0.01 (−0.03, 0.01)	0.180
Poverty income ratio				
Below poverty level (<1.85)	Ref	Ref	Ref	Ref
At or above poverty level (≥1.85)	−0.05 (−0.07, −0.03)	**<0.0001**	0.06 (0.04, 0.07)	**<0.0001**
Body mass index (*n*, %)				
Underweight	−0.03 (−0.09, 0.03)	0.261	−0.03 (−0.08, 0.03)	0.386
Normal weight	Ref	Ref	Ref	Ref
Overweight	0.15 (0.12, 0.17)	**<0.0001**	0.14 (0.12, 0.17)	**<0.0001**
Obese	0.54 (0.52, 0.56)	**<0.0001**	0.53 (0.51, 0.56)	**<0.0001**
HOMA-IR (%)				
Race				
Hispanic	Ref	Ref	Ref	Ref
Non-Hispanic white	−0.09 (−0.12, −0.07)	**<0.0001**	−0.06 (−0.09, −0.04)	**<0.0001**
Non-Hispanic black	−0.04 (−0.06, −0.01)	**0.005**	−0.03 (−0.06, −0.01)	**0.003**
Other races	−0.05 (−0.09, −0.01)	**0.012**	−0.01 (−0.05, 0.03)	0.568
Sex				
Male	Ref	Ref	Ref	Ref
Female	0.05 (0.03, 0.07)	**<0.0001**	−0.01 (−0.03, 0.01)	0.161
Poverty income ratio				
Below poverty level (<1.85)	Ref	Ref	Ref	Ref
At or above poverty level (≥1.85)	−0.04 (−0.06, −0.02)	**<0.0001**	0.04 (0.02, 0.06)	**<0.0001**
Body mass index (n, %)				
Underweight	−0.02 (−0.08, 0.04)	0.450	−0.02 (−0.08, 0.04)	0.555
Normal weight	Ref	Ref	Ref	Ref
Overweight	0.13 (0.11, 0.16)	**<0.0001**	0.13 (0.11, 0.15)	**<0.0001**
Obese	0.52 (0.49, 0.54)	**<0.0001**	0.51 (0.49, 0.54)	**<0.0001**

*Note*: Bold values stand for *p* < 0.05.

Abbreviation: HOMA-IR, homeostasis model assessment-insulin resistance.

^a^Adjusted for race, poverty income ratio, sex, and body mass index.

## Data Availability

The data that support the findings of this study are publicly available in National Health and Nutrition Examination Survey at https://wwwn.cdc.gov/nchs/nhanes/default.aspx, reference number [9].

## Supporting Information

Supporting Information Table S1: Trends in glycometabolic parameters among nondiabetic/nonprediabetic adolescents in the US 1999–2020. Table S2: Differences in glucose and Hemoglobin A1c levels among subgroups. Figure S1: Trends in plasma glucose and hemoglobin A1c levels among sociodemographic subgroups in the US, 1999−2020.
