# Supplementary material for: Trends in Prevalence of Insulin Resistance Among Nondiabetic/Nonprediabetic Adolescents, 1999–2020
Source: Pediatr Diabetes. 2025 May 5;2025:9982025. doi: 10.1155/pedi/9982025 (PMC12069839; doi:10.1155/pedi/9982025)

Supplemental Table 1. Trends in glycometabolic parameters among nondiabetic/non-prediabetic adolescents in the US 1999 to 2020

Characteristics	1999-2000	2001-2002	2003-2004	2005-2006	2007-2008	2009-2010	2011-2012	2013-2014	2015-2016	2017-2020	Relative change per 2-y cycle
	Median (95% CI)	Median (95% CI)	Median (95% CI)	Median (95% CI)	Median (95% CI)	Median (95% CI)	Median (95% CI)	Median (95% CI)	Median (95% CI)	Median (95% CI)	(95% CI)
**Fasting plasma glucose (mg/dl)**											
Overall	89.2 (88.3, 90.2)	91.0 (90.0, 92.1)	89.2 (88.1, 90.3)	89.5 (88.3, 90.7)	90.5 (89.5, 91.6)	89.4 (88.2, 90.6)	89.4 (88.6, 90.2)	89.8 (88.2, 91.4)	90.0 (88.6, 91.4)	91.4 (90.6, 92.1)	0.08 (-0.03, 0.18)
Sex											
Male	90.7 (89.9, 91.5)	92.6 (91.6, 93.7)	90.8 (89.3, 92.2)	91.7 (90.5, 92.9)	91.6 (90.9, 92.3)	90.2 (89.0, 91.5)	90.6 (90.0, 91.2)	92.0 (90.3, 93.8)	92.0 (90.4, 93.6)	92.0 (91.4, 92.7)	0.03 (-0.08, 0.13)
Female	87.8 (86.6, 89.0)	89.3 (88.3, 90.3)	88.1 (86.8, 89.4)	87.8 (86.4, 89.2)	88.7 (87.1, 90.3)	88.2 (87.0, 89.5)	87.3 (85.9, 88.7)	87.9 (86.6, 89.2)	87.8 (86.2, 89.5)	90.4 (89.4, 91.5)	1999-2013: -0.08 (-0.27, 0.11)<br>2013-2020: 0.72 (-0.96, 2.43)
Race											
Hispanic	89.1 (88.2, 90.0)	92.0) (90.3, 93.6)	88.6 (87.0, 90.1)	91.6 (89.7, 93.4)	90.9 (89.4, 92.4)	89.1 (88.1, 90.1)	90.0 (88.7, 91.4)	90.6 (89.2, 91.9)	89.5 (87.7, 91.3)	92.1 (91.3, 93.0)	0.13 (-0.01, 0.27)
Non-Hispanic white	89.7 (88.5, 90.8)	91.3 (90.0), 92.7)	89.6 (88.1, 91.1)	89.2 (87.8, 90.5)	91.0 (89.4, 92.6)	89.1 (88.0, 90.3)	89.8 (89.0, 90.7)	89.9 (87.7, 92.2)	90.2 (88.3, 92.1)	91.2 (90.2, 92.2)	0.05 (-0.07, 0.16)
Non-Hispanic black	87.5 (86.5, 88.4)	87.6 (86.4, 88.8)	87.7 (86.5, 88.9)	88.2 (87.4, 89.0))	87.6 (86.3, 88.9)	87.9 (84.2, 91.6)	86.7 (85.5, 87.9)	87.8 (84.9, 90.6)	88.2 (84.7, 91.6)	89.6 (87.6, 91.6)	0.04 (-0.08, 0.15)
Other Races	89.5 (86.5, 92.4)	90.8 (88.3, 93.4)	90.9 (87.5, 94.3)	91.6 (87.8, 95.4)	90.1 (84.5, 95.6)	92.3 (90.7, 94.0)	86.2 (83.6, 88.9)	90.5 (87.8, 93.1)	91.8 (90.0, 93.7)	91.8 (89.9, 93.8)	0.07 (-0.18, 0.32)
Poverty income ratio											
Below poverty level (<1.85)	89.3 (88.4, 90.1)	90.5 (89.3, 91.6)	88.5 (86.9, 90.1)	89.3 (88.1, 90.5)	91.3 (89.8, 92.8)	89.5 (88.3, 90.8)	88.8 (86.9, 90.7)	89.3 (87.9, 90.8)	90.3 (89.2, 91.3)	91.5 (90.7, 92.3)	0.10 (-0.01, 0.20)
At or above poverty level (⩾ 1.85)	89.4 (88.1, 90.6)	91.3 (89.9, 92.7)	89.7 (88.2, 91.2)	89.7 (88.3, 91.1)	89.9 (88.1, 91.7)	89.0 (88, 90.1)	89.8 (89.0, 90.6)	90.4 (88.0, 92.8)	89.8 (86.9, 92.7)	91.2 (90.2, 92.1)	0.05 (-0.07, 0.17)
**Hemoglobin A1c (%)**											

Overall	5.0 (4.9, 5.0)	5.1 (5.1, 5.1)	5.1 (5.1, 5.1)	5.0 (5, 5)	5.1 (5.1, 5.2)	5.2 (5.1, 5.2)	5.2 (5.1, 5.2)	5.1 (5, 5.2)	5.2 (5.1, 5.2)	5.1 (5.1, 5.2)	0.08 (-0.01, 0.17)
Sex											
Male	5.0 (4.9, 5.0)	5.1 (5.1, 5.2)	5.1 (5.1, 5.2)	5.0 (5.0, 5.0)	5.1 (5.0, 5.2)	5.2 (5.1, 5.2)	5.1 (5.0, 5.2)	5.1 (5, 5.2)	5.2 (5.2, 5.3)	5.1 (5.1, 5.2)	0.08 (-0.07, 0.22)
Female	4.9 (4.9, 5.0)	5.1 (5.0, 5.1)	5.1 (5.0, 5.1)	5.0 (5.0, 5.1)	5.1 (5.0, 5.2)	5.2 (5.1, 5.2)	5.2 (5.2, 5.3)	5.1 (5, 5.2)	5.1 (5.1, 5.2)	5.1 (5.1, 5.2)	0.19 (0.06, 0.33) *
Race											
Hispanic	5.0 (4.9, 5.0)	5.1 (5.0, 5.1)	5.1 (5.0, 5.2)	5.0 (4.9, 5.0)	5.0 (4.9, 5.1)	5.1 (5.1, 5.2)	5.2 (5.1, 5.2)	5.1 (5.1, 5.2)	5.2 (5.1, 5.2)	5.2 (5.1, 5.2)	0.17 (0.05, 0.30)*
Non-Hispanic white	4.9 (4.9, 5.0)	5.1 (5.0, 5.1)	5.1 (5.0, 5.1)	5.0 (4.9, 5.0)	5.1 (5.0, 5.2)	5.2 (5.1, 5.2)	5.1 (5.1, 5.2)	5.0 (4.9, 5.1)	5.1 (5.0, 5.2)	5.1 (5.1, 5.1)	0.11 (-0.03, 0.24)
Non-Hispanic black	5.1 (5.0, 5.2)	5.2 (5.2, 5.3)	5.2 (5.2, 5.3)	5.2 (5.1, 5.3)	5.2 (5.1, 5.2)	5.2 (5.1, 5.3)	5.3 (5.3, 5.4)	5.2 (5.2, 5.3)	5.3 (5.2, 5.4)	5.2 (5.2, 5.3)	0.11 (-0.03, 0.25)
Other Races	5.0 (4.9, 5.1)	5.1 (5.0, 5.2)	5.2 (5.0, 5.3)	5.0 (4.9, 5.1)	5.1 (4.7, 5.6)	5.3 (5.1, 5.4)	5.2 (5.0, 5.4)	5.2 (5.1, 5.3)	5.2 (5.1, 5.3)	5.2 (5.1, 5.3)	0.21 (0.06, 0.35)*
Poverty income ratio											
Below poverty level (<1.85)	5.0 (4.9, 5.0)	5.1 (5.0, 5.1)	5.1 (5.1, 5.2)	5.0 (4.9, 5.1)	5.1 (5.0, 5.2)	5.2 (5.1, 5.2)	5.2 (5.1, 5.2)	5.1 (5.1, 5.2)	5.2 (5.2, 5.3)	5.1 (5.1, 5.2)	0.17 (0.05, 0.29) *
At or above poverty level (⩾ 1.85)	4.9 (4.9, 5.0)	5.1 (5.1, 5.1)	5.1 (5.1, 5.1)	5.0 (5.0, 5.0)	5.1 (5.0, 5.2)	5.2 (5.1, 5.2)	5.2 (5.1, 5.2)	5.1 (5.0, 5.2)	5.1 (5.1, 5.2)	5.1 (5.1, 5.2)	0.14 (-0.02, 0.31)

All estimates are weighted.

* p<0.05, ** p<0.

Supplemental Table 2. Differences in glucose and Hemoglobin A1c levels among subgroups (regression models)

Characteristic	Crude β (95% CI)	p value	Adjusted β (95% CI) *	p value
**Fasting plasma glucose (mg/dl)**				
Race				
Hispanics	Ref	Ref	Ref	Ref
Non-Hispanic white	-0.32 (-0.72, 0.09)	0.122	-0.47 (-0.88, -0.07)	**0.023**
Non-Hispanic black	-2.34 (-2.75, -1.93)	**<.001**	-2.49 (-2.89, -2.09)	**<.001**
Other Races	-0.02 (-0.64, 0.60)	0.948	-0.18 (-0.79, 0.44)	0.575
Sex				
Male	Ref	Ref	Ref	Ref
Female	-2.61 (-2.92, -2.29)	**<.001**	0.02 (-0.31, 0.35)	0.911
Poverty income ratio				
Below poverty level (<1.85)	Ref	Ref	Ref	Ref
At or above poverty level (≥1.85)	0.24 (-0.08, 0.57)	0.143	-2.63 (-2.94, -2.31)	**<.001**
**Hemoglobin A1c (%)**				
Race				
Hispanics	Ref	Ref	Ref	Ref
Non-Hispanic white	-0.03 (-0.04, -0.01)	**0.004**	-0.03 (-0.05, -0.01)	**0.003**
Non-Hispanic black	0.07 (0.05, 0.09)	**<.001**	0.07 (0.05, 0.09)	**<.0001**
Other Races	0.05 (0.02, 0.08)	**<.001**	0.05 (0.02, 0.07)	**0.001**
Sex				
Male	Ref	Ref	Ref	Ref
Female	-0.02 (-0.03, 0)	**0.020**	0.01 (-0.01, 0.02)	0.453
Poverty income ratio				
Below poverty level (<1.85)	Ref	Ref	Ref	Ref
At or above poverty level (≥1.85)	0 (-0.02, 0.01)	0.568	-0.01 (-0.03, 0)	0.082

*Adjusted for race, PIR, and sex.

Supplemental Figure 1. Trends in plasma glucose and hemoglobin A1c levels among sociodemographic subgroups in the US, 1999-2020

Trends in plasma glucose and hemoglobin A1c levels stratified by sex (A), race/ethnicity (B), and poverty index ratio (C). Error bars indicate 95% CIs. P values for trends and annual percent change (APC) were obtained from joinpoint regressions. Significant upward trends in hemoglobin A1c were only observed among the subgroups of girls, Hispanic, other race and low PIR. Specific estimates are presented in Supplemental Table 1. PIR, poverty income ratio. * p<0.05, ** p<0.01

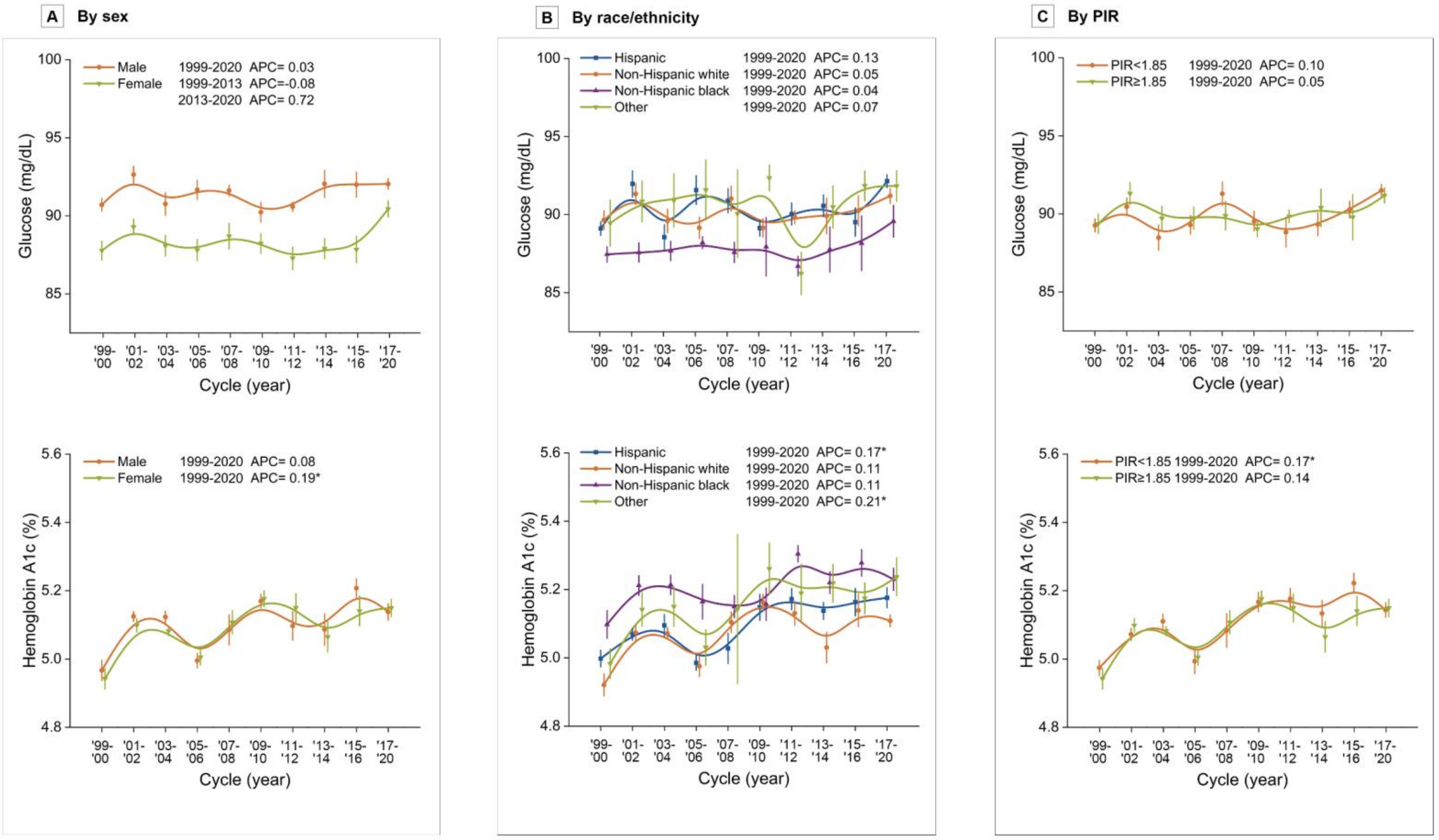

Supplement: Supporting Information — Table S1: Trends in glycometabolic parameters among nondiabetic/nonprediabetic adolescents in the US 1999–2020. Table S2: Differences in glucose and Hemoglobin A1c levels among subgroups. Figure S1: Trends in plasma glucose and hemoglobin A1c levels among sociodemographic subgroups in the US, 1999−2020. [file 9982025.f1.pdf]
